# Investigation of the Distribution of *Salmonella* within an Integrated Pig Breeding and Production Organisation in the United Kingdom

**DOI:** 10.1155/2013/943126

**Published:** 2013-12-17

**Authors:** A. Wales, J. Weaver, I. M. McLaren, R. P. Smith, D. Mueller-Doblies, R. H. Davies

**Affiliations:** ^1^Department of Bacteriology and Food Safety, Surveillance and Risk Group, Animal Health and Veterinary Laboratories Agency, Woodham Lane, New Haw, Addlestone, Surrey KT15 3NB, UK; ^2^Australia-Indonesia Partnership for Emerging Infectious Diseases, Ministry of Agriculture, Jakarta 12550, Indonesia; ^3^Epidemiology, Surveillance and Risk Group, Animal Health and Veterinary Laboratories Agency, Woodham Lane, New Haw, Addlestone, Surrey KT15 3NB, UK

## Abstract

To examine patterns of *Salmonella* herd infections in units linked by common sources of pigs, the study examined pooled pen faeces samples from 161 nursery and finishing units in a UK integrated pig enterprise. An epidemiological questionnaire was also completed by investigators for each farm. *Salmonella* was isolated from 630 (19.5%) of the samples: *S. *Typhimurium was found in 387 (12%) and *S.* Derby in 157 (4.9%) samples; 111 units yielded at least one sample containing *Salmonella*. The proportion of *Salmonella*-positive samples from positive farms ranged from 5% to 95%. In a univariable risk factor analysis, increasing length of time as a pig farm was positively associated with the detection of *Salmonella* in a herd. Larger farms (>500 pigs) were significantly more likely to be positive for *S. *Typhimurium than smaller farms. There was an association between *Salmonella* serovars isolated in the present study and those subsequently isolated in breeding herds linked to the integration.

## 1. Introduction

Recent European attribution studies have implicated pork as a principal source of human salmonellosis [1, 2], and minimising the carriage of *Salmonella* by slaughter-age pigs is considered to be a useful control for *Salmonella* contamination on carcasses [3–5]. The large herds of modern pig production [6], plus movements of pigs between premises at different life stages, offer opportunities for the maintenance and dissemination of *Salmonella* infections that may originate in breeding stock or be acquired from endemic environmental contamination of premises [7, 8]. On-farm risk factor analyses have commonly identified associations between herd *Salmonella* status or individual infections and feed type, hygiene, biosecurity, contact between pig groups, number of suppliers, previous clinical salmonellosis, and certain intercurrent diseases [9].

Vertically integrated pig companies control sequential production levels, from genetic or multiplier breeding herds to slaughter of finished pigs. They may use external contractors to implement commercial breeding and finishing [10], moving pigs between contractors' sites between life stages. Such integrated pig production systems may potentially offer more control over sources of *Salmonella* infection, but the risks of dissemination of infection that is present in the upper tiers of the production pyramid may also be substantial.

The present report describes a study examining *Salmonella* contamination on multiple sites that were part of a single integration sharing a common breeding source. Pooled faeces sampling coupled with sensitive culture-based detection [11] allowed cost-effective and reliable isolation of typable strains. The study aimed to determine the extent of *Salmonella *infection across sites contracted to this pig integration and, through the use of a questionnaire, to seek associated risk factors.

The study was performed some time ago (in the late 1990s) and reported as part of a UK strategic *Salmonella* research project (OZ0134). However, given the ongoing issues with *Salmonella* (and *Salmonella* Typhimurium (STM) plus monophasic variants in particular) in pig breeding and production herds [12, 13], the authors consider that the data may be of interest to a wider audience.

## 2. Materials and Methods

The integration used contracted sites in England operating a genetic multiplier herd plus commercial breeding, indoor nursery, and finishing units, the last two accommodating growing pigs of around four to 10 weeks and 10 to 23 weeks, respectively. Replacement genetic stock was obtained from a major primary breeding company. Fattening and breeding units were operated on a single-age all-in-all-out basis. The main study was restricted to those nursery and finishing units that had consented to participate although a small number of breeding herds were sampled independently by the research team.

Within an 18 month period each participating farm had a single veterinary visit during which naturally pooled faeces (10–15 g per pool) were collected from each of 20 pens, randomly selected from a sample frame of all houses on the farm, or from all pens if there were fewer than 20. Faeces were collected into sterile jars by hand using clean single-use latex gloves and samples were packed and shipped by post on the day of collection and at ambient temperature to the Veterinary Laboratories Agency (VLA) Weybridge. At the same visit an epidemiological questionnaire, containing sections on demography, farm structure and management details, herd details, antibiotic therapy, disease security, and hygiene measures was completed. This was sufficient to generate data for a list of candidate risk factors (Table 1). A specimen questionnaire is available on request from the authors. Notes were also made following a visual assessment of general farm hygiene, including cleaning and disinfection (C&D).

Samples were cultured for *Salmonella *using a sensitive technique for environmental samples utilising pre-enrichment in buffered peptone water, enrichment on semisolid (DIASSALM) agar, and detection on selective indicator (Rambach) agar, as previously described [14]. Representative suspect colonies of *Salmonella *were screened using polyvalent O and H antisera and were later subjected to full serotyping in the *Salmonella *reference laboratory at VLA Weybridge.

A univariable analysis was performed for associations between categorical or continuous questionnaire variables (Table 1) and the presence of any *Salmonella* on the farm, the presence of STM on the farm, and the proportion of *Salmonella-*positive samples from the farm. Chi-squared and Student's *t*-tests were used, completed using Epi Info 6 (http://wwwn.cdc.gov/epiinfo/). Owing to the low values in some of the categories analysed by Chi-squared, Yates's correction was applied and where an expected value was less than 5, Fisher exact *P* values were applied.

## 3. Results and Discussion

One hundred and sixty one farms agreed to participate. Details of farm types and herd sizes are provided in Table 2. The modal numbers of pigs per farm in the study are close to average (mean) values for commercial fattening pig units in England from recent years [15].

*Salmonella* and STM were isolated from 19.5% and 12%, respectively, of 3220 samples collected. On each of the 111 (68.9%) *Salmonella-*positive farms, between 5% and 95% of samples yielded *Salmonella*. Among *Salmonella*- and STM-positive units, those with over 50% positive samples were more frequent in the nursery category (28% and 17% resp.) than in the finisher category (13% and 7% resp.). *P* values for these differences (Yates correction for both, Fisher exact test for STM) did not achieve significance, being 0.094 and 0.175, respectively. Nearly half of the farms yielded more than one serovar: two were found on 28 farms, three on 14 farms, four on five farms, and five on three farms. Tables 3 and 4 provide further details for all identified serovars, and details of isolations by farm category (nursery and finisher) are given in Table 2.

More recent studies, using similar sampling methodologies in finishing pig herds in Canada and Spain, have reported herd-level prevalence between 12% and 58% [16–19]. Therefore, the nursery and finisher herd-level prevalence in the present report sit a little above the upper end of results obtained more recently elsewhere. Nonetheless, the observed predominance of STM is consistent with more recent UK pig reports [13, 20–22] and the findings of other European investigations [17, 22–26]. The wide variation seen in the within-herd proportion of positive samples is consistent with other cross-sectional and longitudinal studies [19, 21, 24, 27] and may reflect the dynamic nature of *Salmonella* infections among growing pigs.

The risk factor study was limited, by the resources available, to a univariable analysis. For many chosen pairings of risk factor and outcome the univariable analysis was found to be not valid, owing to insufficient data within the different categories.

There was a significant positive association between finding *Salmonella* on a farm and the length of time that unit had been a pig farm (Table 5). Of the farms that had kept pigs for five or fewer years, 58.1% were *Salmonella*-positive, compared with 88.5% of farms that had kept pigs for more than 30 years (*P* = 0.025, Yates corrected). Farms with *Salmonella* had kept pigs for significantly longer (mean 22.7 years) than farms without *Salmonella* (16.6 years; *t*-test, *P* = 0.023). Similarly, 39% of farms that had kept pigs for five or fewer years were STM positive compared with 69% of farms which had kept pigs for over 30 years (Table 5), which was a significant difference (*P* = 0.042, Yates corrected).

This “time as a pig farm” variable may act as an indicator for a cluster of risk factors that affect susceptibility to endemic *Salmonella* contamination, for example, the types and conditions of buildings and their materials and the design and organisation of the farm for biosecurity, batch management, and hygiene. It may also be that long-established farms have had more opportunities to acquire endemic *Salmonella* strains. Evidence of endemic persistence of particular strains includes the presence over several years of the distinctive serovar *S.* Panama on some of the farms [28].

The other significant associations identified were between the size of herd and the presence of STM specifically. This was the case for both nursery herds (>500 pigs, 67.3% herds positive; <500 pigs 20.0% herds positive: *P* = 0.011 Yates corrected and Fisher exact *P* value) and for finishing herds (equivalent statistics 62.7% versus 32.0%; *P* = 0.015, Yates corrected).

More complex multivariable modelling approaches [29–31] have also identified similar variables, of herd size or production volume, as risk factors for *Salmonella* shedding. Reasons why the larger herds may have had a higher risk of being *Salmonella* positive include acquisition policies (especially multiple sources) and herd immunity effects such as nonuniform exposure and small numbers of pigs with a poor immune response, which shed *Salmonella* heavily. These effects may act preferentially in larger herds to sustain cycles of infection.

It is uncertain whether the observed difference between STM and other serovars in respect of an association with herd size is genuine. However, evidence from elsewhere [32, 33] suggests that infections with STM may be more long lasting in growing pigs than those with other serovars, which may interact with herd immunity effects discussed above to reduce the likelihood that infections will spontaneously recede or resolve in larger herds.

A poor standard of C&D was noted on some farms although quantitative assessment of its efficacy was not performed. Furthermore, the concentration of disinfectant used on the majority of units was found to be approximately half that of the MAFF (Defra) approved General Orders rate under the Animal Health Act (1981). Several other studies have reported the efficacy of C&D for removing *Salmonella *on pig farms to be poor [20, 21, 27, 34], and measures of poor C&D have been identified in some analyses as risk factors for positive *Salmonella* status [29, 35].

Breeding herds were not included in the present survey, mostly for logistic reasons, but the importance of breeding herd sources to *Salmonella* in pig production has been firmly established by other investigations [21, 36, 37]. Furthermore, subsequent studies of the present company's multiplier herd and commercial breeding herds have identified the same range of serovars that were found in the nursery and finishing farms [28]. This included the unusual UK pig serovar* S.* Panama, and is consistent with the spread of *Salmonella* throughout the integration. In addition, new gilts received from a primary breeding company were found to carry STM [21], indicating a potential route for introduction of STM from higher up in the breeding pyramid.

In conclusion, the findings suggest that vertical integration does not necessarily achieve superior control of *Salmonella* in the supply and fattening of pigs (compared with independent fattening or farrow-to-finish units) despite the theoretical advantages of integrated management in this respect. On the present evidence, significant challenges to *Salmonella* control in an integration may exist simultaneously in both the breeding pyramid (a route for *Salmonella* entering production herds) and in individual production units, where imported or environmental *Salmonella* needs to be controlled and eliminated.

## Figures and Tables

**Table 1:** Potential risk factors analysed from questionnaire.

Housing	Management	Biosecurity/hygiene
Type of pen (slatted, push-through, straw yard)	Number of staff Number of pigs on farm Time as a pig farm Age groups housed separately Feed source Sick pens Reintegration of recovered pigs Bedding source Bedding type Feeder/drinker systems	Staff contact with other pigs Visitors who have contact with other pigs Water supply (mains or borehole) Drainage Presence of wildlife species Proximity of waterways, scrubland, pig wastes, cattle wastes, or sewage treatment/landfill sites Feed storage (capacity and sealed/open bins) Presence of biosecurity measures (boot dips, wheel wash, and visitor/staff clothing) Pigs loaded at perimeter Site contained within perimeter fence Rodent controls Disinfectant: type and frequency

**Table 2:** Number and size of pig farms in the study and their *Salmonella* status.

Pig category	Herd size	Number of farms sampled	Number of farms *Salmonella-*positive	
			Any serovar	*S. *Typhimurium
Nursery	<201	0	0	0
	201–500	10	5	2
	501–800	13	11	9
	801–1100	6	6	6
	1101–1400	11	7	6
	1401–1700	7	7	7
	1701–2000	6	3	2
	>2000	6	4	3
Nursery totals		**59**	**43**	**35**

Finishing	<201	6	3	2
	201–500	19	11	6
	501–800	15	10	9
	801–1100	19	12	10
	1101–1400	14	10	8
	1401–1700	7	5	4
	1701–2000	6	5	5
	>2000	14	11	11
	Unknown	2	1	1
Finishing totals		**102**	**68**	**56**

**Table 3:** *Salmonella* serovars and numbers of isolates.

Serovar	Number isolated	Percentage of isolates
*S. *Typhimurium	387	61.4
*S. *Derby	157	24.9
*S. *Panama	31	4.9
*S. *Goldcoast	19	3.0
*S. *Reading	14	2.2
*S. *London	5	0.8
*S. *Anatum	4	0.6
*S. * 4,12:-:-	3*	0.5
*S. *Agona	2	0.3
*S. *Manhattan	2	0.3
*S. *Enteritidis	1	0.2
*S. *Brandenburg	1	0.2
*S. *Bovismorbificans	1	0.2
*S. *Kentucky	1	0.2
*S. *Kimuenza	1	0.2
*S. *Schwarzengrund	1	0.2

*As the study was conducted before the emergence of monophasic strains of *S*. Typhimurium in pigs in the UK, no such isolates were found. However, three isolates of an aphasic group B strain were identified.

**Table 4:** Extent of *Salmonella* infection on study farms.

Percent positive samples	Number of farms in category	
	*S. *Typhimurium (STM)	Non-STM *Salmonella *
0*	70 (43.5%)	97 (60.2%)
5–25	68 (42.2%)	51 (31.7%)
30–50	13 (8.1%)	10 (6.2%)
55–75	8 (5.0%)	1 (0.6%)
80–100	2 (1.2%)	2 (1.2%)

*Values in this row indicate the number (and percentage) of farms yielding no isolates of either STM or non-STM *Salmonella*. Fifty out of the 161 farms did not yield any *Salmonella* isolates at all.

**Table 5:** Comparison of the length of time that a farm had kept pigs against isolation of *Salmonella*.

*Salmonella* isolates	Time that farm had been used for pigs (years)						Total farms
	5 or less	6–10	11–20	21–30	30+	Unknown	
None	13 (26%)	12 (24%)	8 (16%)	14 (28%)	3 (6%)	0 (0%)	50
Any serovar	18 (16%)	16 (14%)	22 (20%)	30 (27%)	23 (21%)	2 (2%)	111
*S. *Typhimurium	12 (13%)	15 (16%)	19 (21%)	27 (29%)	18 (20%)	1 (1%)	91

Principal values are the number of study farms in each category. Values in parentheses are the percentage for that *Salmonella* isolation category.
